# Supplementary material for: The Impact of Multiple Sclerosis Disease Status and Subtype on Hematological Profile
Source: Int J Environ Res Public Health. 2021 Mar 23;18(6):3318. doi: 10.3390/ijerph18063318 (PMC8004915; doi:10.3390/ijerph18063318)
Supplement: Supplementary file 1 [file ijerph-18-03318-s001.zip › IJERPH S1.docx]

| Supplementary Table 1. Number of patients in each case-control analysis | | | | | |
| --- | --- | --- | --- | --- | --- |
|  | Caucasian | |  | African American | |
|  | Case | Control |  | Case | Control |
| **CBC** |  |  |  |  |  |
| Hemoglobin (Hgb) | 4191 | 36071 |  | 585 | 3243 |
| Immature platelet fraction (IPF) | 115 | 852 |  | 23 | 120 |
| Immature reticulocyte fraction (IRF) | 138 | 1196 |  | 25 | 165 |
| Lymphocyte absolute count (LymAbs) | 3815 | 20041 |  | 552 | 1938 |
| Mean corpuscular hemoglobin (MCH) | 4187 | 36066 |  | 585 | 3243 |
| MHC concentration (MCHC) | 4187 | 36066 |  | 585 | 3243 |
| Mean corpuscular volume (MCV) | 4188 | 36067 |  | 585 | 3243 |
| Mean platelet volume (MPV) | 2768 | 20803 |  | 396 | 1840 |
| Neutrophil absolute count (NeutAbs) | 3814 | 25074 |  | 552 | 2410 |
| Neutrophil-to-lymphocyte ratio (NLR) | 3814 | 20017 |  | 552 | 1936 |
| Packed cell volume (PCV) | 4299 | 38513 |  | 588 | 3420 |
| Platelet count (PltCt) | 4173 | 36168 |  | 579 | 3250 |
| Red blood cell count (RBC) | 4188 | 36068 |  | 585 | 3243 |
| Red cell distribution width (RDW) | 4187 | 36061 |  | 585 | 3242 |
| RDW standard deviation (RDWSD) | 2912 | 28888 |  | 419 | 2565 |
| Reticulocytes absolute value (RetAbs) | 204 | 1617 |  | 37 | 238 |
| Reticulocyte Hgb equivalent (RETHE) | 114 | 835 |  | 23 | 116 |
| Reticulocyte count (RetiCt) | 204 | 1616 |  | 37 | 237 |
| White blood cell count (WBC) | 4192 | 36071 |  | 585 | 3243 |
| **CMP** |  |  |  |  |  |
| Albumin (Alb) | 3735 | 25557 |  | 540 | 2130 |
| Alkaline phosphatase (AlkP) | 3855 | 26803 |  | 554 | 2338 |
| Anion gap (ANGAP) | 3229 | 36063 |  | 449 | 3110 |
| Blood urea nitrogen (BUN) | 3423 | 36157 |  | 482 | 3121 |
| Calcium (Ca) | 3339 | 35957 |  | 468 | 3101 |
| Chloride (Cl) | 3369 | 36163 |  | 474 | 3122 |
| Carbon dioxide (CO2) | 3371 | 36190 |  | 474 | 3124 |
| Creatinine (Creat) | 3458 | 36396 |  | 484 | 3149 |
| Glucose (Gluc) | 3379 | 36238 |  | 474 | 3137 |
| Icterus index (IctIdx) | 178 | 1959 |  | 14 | 117 |
| Potassium (K) | 3375 | 36203 |  | 474 | 3124 |
| Lipid index (LipIdx) | 213 | 2085 |  | 20 | 135 |
| Sodium (Na) | 3372 | 36200 |  | 474 | 3125 |
| Aspartate amino transferase (SGOT) | 3886 | 27215 |  | 559 | 2387 |
| Alanine amino transferase (SGPT) | 3867 | 27020 |  | 556 | 2353 |
| Bilirubin (TBil) | 3841 | 26833 |  | 552 | 2332 |
| Total protein (TProt) | 3753 | 25387 |  | 540 | 2108 |
