# Supplementary material for: The Impact of Multiple Sclerosis Disease Status and Subtype on Hematological Profile
Source: Int J Environ Res Public Health. 2021 Mar 23;18(6):3318. doi: 10.3390/ijerph18063318 (PMC8004915; doi:10.3390/ijerph18063318)
Supplement: Supplementary file 1 [file ijerph-18-03318-s001.zip › IJERPH S2.docx]

| Supplementary Table 2. Number of patients in each Caucasian cohort subtype analysis | | | | |
| --- | --- | --- | --- | --- |
|  | PPMS (M) | PPMS (F) | RRMS/SPMS (M) | RRMS/SPMS (F) |
| **CBC** |  |  |  |  |
| Hemoglobin (Hgb) | 82 | 172 | 376 | 1248 |
| Immature platelet fraction (IPF) | 1 | 3 | 5 | 22 |
| Immature reticulocyte fraction (IRF) | 2 | 5 | 7 | 29 |
| Lymphocyte absolute count (LymAbs) | 77 | 163 | 364 | 1201 |
| Mean corpuscular hemoglobin (MCH) | 82 | 172 | 375 | 1248 |
| MHC concentration (MCHC) | 82 | 172 | 375 | 1248 |
| Mean corpuscular volume (MCV) | 82 | 172 | 375 | 1248 |
| Mean platelet volume (MPV) | 50 | 107 | 256 | 812 |
| Neutrophil absolute count (NeutAbs) | 77 | 163 | 364 | 1200 |
| Neutrophil-to-lymphocyte ratio (NLR) | 77 | 163 | 364 | 1200 |
| Packed cell volume (PCV) | 83 | 176 | 378 | 1264 |
| Platelet count (PltCt) | 82 | 171 | 374 | 1247 |
| Red blood cell count (RBC) | 82 | 172 | 375 | 1248 |
| Red cell distribution width (RDW) | 82 | 172 | 375 | 1248 |
| RDW standard deviation (RDWSD) | 51 | 112 | 250 | 839 |
| Reticulocytes absolute value (RetAbs) | 4 | 8 | 11 | 45 |
| Reticulocyte Hgb equivalent (RETHE) | 1 | 3 | 5 | 22 |
| Reticulocyte count (RetiCt) | 4 | 8 | 11 | 45 |
| White blood cell count (WBC) | 82 | 172 | 375 | 1249 |
| **CMP** |  |  |  |  |
| Albumin (Alb) | 79 | 158 | 361 | 1192 |
| Alkaline phosphatase (AlkP) | 80 | 161 | 365 | 1201 |
| Anion gap (ANGAP) | 67 | 116 | 256 | 841 |
| Blood urea nitrogen (BUN) | 71 | 132 | 283 | 903 |
| Calcium (Ca) | 68 | 121 | 271 | 877 |
| Chloride (Cl) | 70 | 122 | 275 | 889 |
| Carbon dioxide (CO2) | 70 | 122 | 275 | 889 |
| Creatinine (Creat) | 71 | 134 | 284 | 909 |
| Glucose (Gluc) | 69 | 122 | 274 | 888 |
| Icterus index (IctIdx) | 5 | 9 | 19 | 53 |
| Potassium (K) | 70 | 122 | 275 | 890 |
| Lipid index (LipIdx) | 5 | 9 | 19 | 53 |
| Sodium (Na) | 70 | 122 | 275 | 889 |
| Aspartate amino transferase (SGOT) | 80 | 162 | 367 | 1208 |
| Alanine amino transferase (SGPT) | 80 | 162 | 366 | 1202 |
| Bilirubin (TBil) | 80 | 161 | 365 | 1197 |
| Total protein (TProt) | 79 | 161 | 362 | 1188 |
| M = male; F = female | | | | |
