# Supplementary material for: The Impact of Multiple Sclerosis Disease Status and Subtype on Hematological Profile
Source: Int J Environ Res Public Health. 2021 Mar 23;18(6):3318. doi: 10.3390/ijerph18063318 (PMC8004915; doi:10.3390/ijerph18063318)
Supplement: Supplementary file 1 [file ijerph-18-03318-s001.zip › IJERPH S3.docx]

| Supplementary Table 3. Number of patients in each African-American cohort subtype analysis | | | | |
| --- | --- | --- | --- | --- |
|  | PPMS (M) | PPMS (F) | RRMS/SPMS (M) | RRMS/SPMS (F) |
| **CBC** |  |  |  |  |
| Hemoglobin (Hgb) | 14 | 32 | 36 | 188 |
| Immature platelet fraction (IPF) | 2 | 2 | 2 | 6 |
| Immature reticulocyte fraction (IRF) | 2 | 2 | 2 | 7 |
| Lymphocyte absolute count (LymAbs) | 15 | 29 | 35 | 185 |
| Mean corpuscular hemoglobin (MCH) | 14 | 32 | 36 | 188 |
| MHC concentration (MCHC) | 14 | 32 | 36 | 188 |
| Mean corpuscular volume (MCV) | 14 | 32 | 36 | 188 |
| Mean platelet volume (MPV) | 14 | 17 | 26 | 127 |
| Neutrophil absolute count (NeutAbs) | 15 | 29 | 35 | 185 |
| Neutrophil-to-lymphocyte ratio (NLR) | 15 | 29 | 35 | 185 |
| Packed cell volume (PCV) | 14 | 32 | 36 | 189 |
| Platelet count (PltCt) | 14 | 32 | 36 | 187 |
| Red blood cell count (RBC) | 14 | 32 | 36 | 188 |
| Red cell distribution width (RDW) | 14 | 32 | 36 | 188 |
| RDW standard deviation (RDWSD) | 12 | 19 | 25 | 133 |
| Reticulocytes absolute value (RetAbs) | 2 | 3 | 2 | 12 |
| Reticulocyte Hgb equivalent (RETHE) | 2 | 2 | 2 | 6 |
| Reticulocyte count (RetiCt) | 2 | 3 | 2 | 12 |
| White blood cell count (WBC) | 14 | 32 | 36 | 188 |
| **CMP** |  |  |  |  |
| Albumin (Alb) | 14 | 31 | 35 | 186 |
| Alkaline phosphatase (AlkP) | 14 | 31 | 36 | 186 |
| Anion gap (ANGAP) | 13 | 21 | 22 | 152 |
| Blood urea nitrogen (BUN) | 13 | 25 | 24 | 161 |
| Calcium (Ca) | 13 | 21 | 22 | 158 |
| Chloride (Cl) | 13 | 21 | 23 | 161 |
| Carbon dioxide (CO2) | 13 | 21 | 23 | 161 |
| Creatinine (Creat) | 13 | 26 | 24 | 162 |
| Glucose (Gluc) | 13 | 21 | 23 | 159 |
| Icterus index (IctIdx) | 1 | 2 | 1 | 5 |
| Potassium (K) | 13 | 21 | 23 | 161 |
| Lipid index (LipIdx) | 1 | 2 | 1 | 5 |
| Sodium (Na) | 13 | 21 | 23 | 161 |
| Aspartate amino transferase (SGOT) | 14 | 31 | 36 | 188 |
| Alanine amino transferase (SGPT) | 14 | 31 | 36 | 186 |
| Bilirubin (TBil) | 14 | 31 | 35 | 186 |
| Total protein (TProt) | 14 | 31 | 35 | 187 |
| M = male; F = female | | | | |
